# A review of multistate modelling approaches in monitoring disease progression: Bayesian estimation using the Kolmogorov-Chapman forward equations

**DOI:** 10.1177/0962280221997507

**Published:** 2021-04-07

**Authors:** Zvifadzo Matsena Zingoni, Tobias F. Chirwa, Jim Todd, Eustasius Musenge

**Affiliations:** 1 Division of Epidemiology and Biostatistics, School of Public Health, Faculty of Health Sciences, University of the Witwatersrand, Johannesburg, South Africa; 2 National Institute of Health Research, Causeway, Harare, Zimbabwe; 3 Department of Population Health, London School of Hygiene and Tropical Medicine, London, United Kingdom

**Keywords:** Bayesian estimation, frequentist (maximum likelihood) estimation, Kolmogorov-Chapman forward equations, multistate models, partially observed aggregated data, WinBUGS

## Abstract

There are numerous fields of science in which multistate models are used, including biomedical research and health economics. In biomedical studies, these stochastic continuous-time models are used to describe the time-to-event life history of an individual through a flexible framework for longitudinal data. The multistate framework can describe more than one possible time-to-event outcome for a single individual. The standard estimation quantities in multistate models are transition probabilities and transition rates which can be mapped through the Kolmogorov-Chapman forward equations from the Bayesian estimation perspective. Most multistate models assume the Markov property and time homogeneity; however, if these assumptions are violated, an extension to non-Markovian and time-varying transition rates is possible. This manuscript extends reviews in various types of multistate models, assumptions, methods of estimation and data features compatible with fitting multistate models. We highlight the contrast between the frequentist (maximum likelihood estimation) and the Bayesian estimation approaches in the multistate modeling framework and point out where the latter is advantageous. A partially observed and aggregated dataset from the Zimbabwe national ART program was used to illustrate the use of Kolmogorov-Chapman forward equations. The transition rates from a three-stage reversible multistate model based on viral load measurements in WinBUGS were reported.

## 1 Introduction

A multistate model is defined as a continuous-time stochastic process which allows participants to move among a finite discrete number of compartments or states which could be clinical symptoms, biological markers, disease stages or disease recurrence in biomedical research (1,2). Multistate models are useful in infectious disease monitoring programs which aim to gain an in-depth understanding of the disease progression patterns. Commonly for these models, the disease state is partitioned into a finite number of intermediate states which may offer greater insight and understanding of the disease evolution (2).

Movement from one state to another is called a transition (event has occurred); states can be transient (if a transition can emerge from the state) or absorbing (if no transition can emerge from the state). Movement between transitions can be reversible or irreversible, and these movements contribute to the intricacy of the multistate model in addition to the number of states defined. The transition intensities (hazard rates) provide the transition specific hazards for movement from one discernible state to another. These transition intensity functions can also be used to compute the mean sojourn time (the average time spent in a pre-clinical state before a clinical outcome of interest occurs), the total length of stay in a state (total time spent in a state before making a transition), the number of transitions made from start to end of the study and the transition probabilities. (3). Also, the effect of covariates on each transition can be assessed to quantify the influence of the covariate on the different model transitions. The covariates effects may not be the same since the severity of the disease progression differ by each intermediate state. (4).

**Figure 1:**
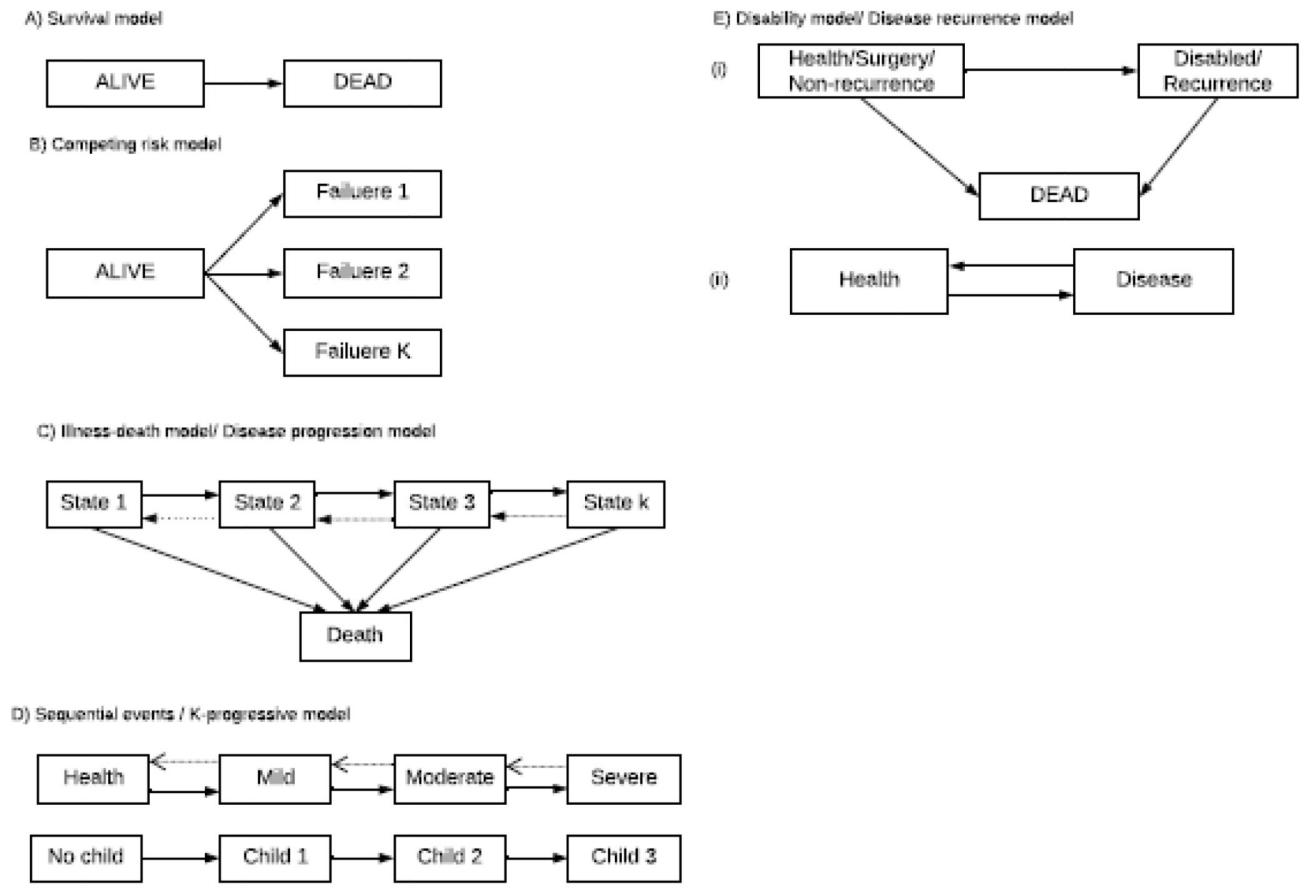
Schematic illustration of different types of multistate models.

There are different types of multistate models which can be used to answer different research questions, Figure 1. The *mortality model* for survival analysis with only two states and one transition from “alive” state to “dead” state is the simplest multistate model, Figure 1(a). These mortality models are useful, mostly in answering etiological research questions(5). The hazard rates are usually estimated using a semi-parametric approach which has a less stringent assumption(6). The hazard function is assumed to be an arbitrary, unspecified, non-negative function of time(7). The incidence or hazard rate is estimated by assuming independence of survival times between distinct individuals in a sample and a constant hazard ratio regardless of ties, especially if the survival time is not discrete(6).

Another type of multistate model is the competing risks model which extends the mortality model depicting a scenario whereby an individual may experience one of the several failure outcomes (8,9), Figure 1(b). In such models, competing risk analysis is performed whereby the interest is in the occurrence of the primary outcome but other contesting events may preclude the occurrence of the primary outcome or significantly alter the chances of observing the primary outcome. Competing risk analysis may also be performed in situations where the different types of events may be relevant, but the analysis focuses on both time and occurrence of the first event(10). The reason why the competing risk analysis is considered to be appropriate over the Kaplan−Meier estimation in such situations described above is that the Kaplan−Meier estimation treats the competing events as censored observations which bring in bias since the independence assumption is violated. To be more explicit, the censoring of the time to primary event by the competing risk event may not be independent of the unobserved time to the primary event. As a result, the baseline hazard may differ between these competing events.11 The competing risk models provide in-depth insight into the effect of interventions on separate outcomes observed. The competing risk models are useful in exploring the relationship between explanatory covariates and the absolute risk which is critical particularly in decision-making and prognostic research work(5).

Partitioning the “alive” state of the mortality model into two or more transient (intermediate) states yields another type of a multistate model known as the *disease progressive multistate model* of which the simplest is the three-state model (2), Figure 1(c). In biomedical research, *illness-death models* or *disability models* which are a special type of a disease progression model, are usually used in estimating disease incidence rates and the mortality transition intensities(12). The disability model is considered in irreversible models when the disease increases the risk of death. In scenarios whereby the absorbing state is not considered, the models are termed *K-progressive models* which follow a sequential process, for instance, health, mild, moderate and severe states with a possibility of reversible transitions (12). However, in some instances, the models might not allow reversible transitions like the *fertility model* which is used to describe the reproductive life history of a woman where each state is defined by the number of children born, Figure 1(d).

The application of multistate models is not limited to biomedical studies like the evaluation of disease progression patterns(13–15) but cuts across various life history data, including health economics. In health economics studies inclined to the monitoring of disease progression, issues on the cost-effectiveness of prevention strategies(16), treatment(17), and infectious disease diagnosis interventions like HIV (18) to inform various policy decision-making processes in HIV control programs (19), can be addressed using multistate models.

There is an extensive review of multistate models in the literature. However, most review papers have focused on the frequentist or maximum likelihood estimation (MLE) approach within the multistate model framework(1,2,4). None of these reviews has discussed in detail the Bayesian estimation (BE) approach within the multistate model framework of which BE approach is equally a robust method in statistical modelling. Therefore, this article aims to extend previous reviews on multistate models with primary emphasis on BE in multistate models. An illustration on the use of Kolmogorov−Chapman forward equations application on partially observed aggregated viral load data is provided.

**Figure 2:**
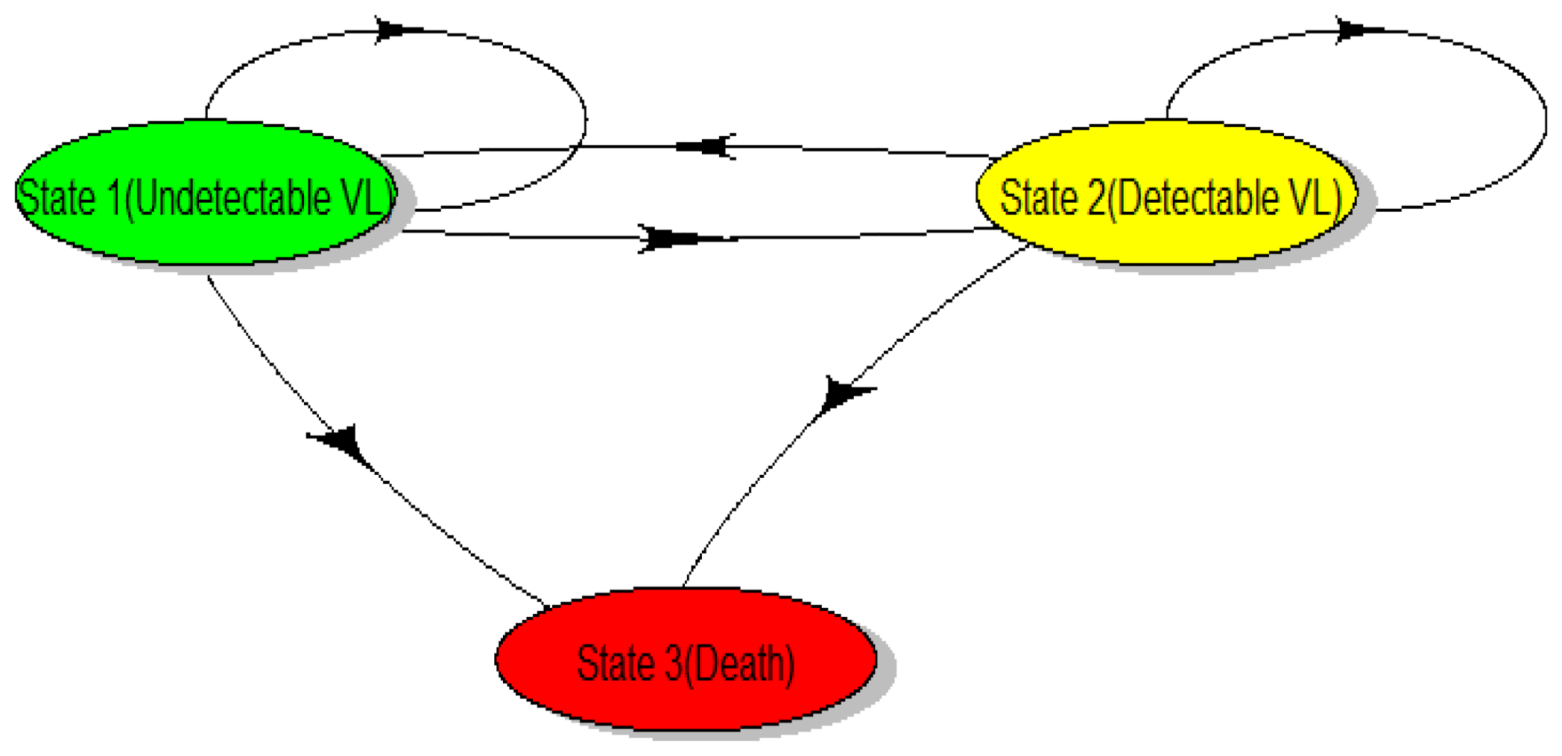
The schematic presentation of three states partially observed multistate model and the corresponding individual-specific transition intensities (State 1=Viral load <50 copies/mL (undetectable); State 2=Viral load ≥50 copies/mL(detectable))

The rest of the manuscript is structured as follows: Section 2 will introduce the theoretical aspects of the MLE approach and BE approach based on the Kolmogorov𢈒Chapman forward equations. This section will integrate the different assumptions within multistate models’ framework, data features and contrast between MLE and BE methods. Section 3 will provide a detailed application on the use of Kolmogorov-Chapman forward equations on partially observed aggregated viral load data. The data in context were extracted from the Zimbabwe national ART programme through the electronic patient management database (ePMS); hence, a sample of patients with viral load measurements measured within a year was used. For this illustration, a three-stage reversible multistate model was considered, with states defined based on viral load measurements, and a schematic presentation of the diagram is shown in Figure 2.

Section 4 is left for discussion and conclusion of the manuscript. The appendix section provides additional supporting information for Section 2 in addition to the Windows version of Bayesian inference Using Gibbs Sampling (WinBUGS) code used.

## 2 Modelling approaches

### 2.1 The Maximum Likelihood Estimation (MLE) Approach

The frequentist approach has been well documented in the literature(1,2). This method strongly relies on the dataset for parameter estimation. In the frequentist approach, the statistical inference and estimation of the transition rates are based on the MLE. To have some in-depth understanding of this modelling approach, detailed theoretical steps behind the estimation of transition rates have been outlined. With reference to the three-stage multistate model shown in Figure 2, the aim is to estimate the four transition parameters shown in the diagram. For the MLE approach, the first step is to get the product of the distribution function of the parameters. Considering an exponential distribution function for the transition rate

(1) $$f\;\left(\gamma_{jk}\left(t_{j}\right)\right)=\frac{{\left(\gamma_{jk}\left(t_{j}\right)\right)}^{n_{jk}}\;\exp\left(-\gamma_{jk}\left(t_{j}\right)\right)}{n_{jk}!}\;\text{for}\;j\;\neq \;k$$

where *γ_jk_* is the transition rate from state *j* to state *k*. The number of the observed movements between the states is represented by *n_jk_*, and *t_j_* is the total observed waiting time in the state *j* for *j* = 1,2. The likelihood function is:

(2) $$L\left(\gamma_{jk}\left(t_{j}\right)\right)=\prod_{j\neq k}\left[\frac{{\left(\gamma_{jk}\left(t_{j}\right)\right)}^{n_{jk}}\text{exp}\left(-\gamma_{jk}\left(t_{j}\right)\right)}{n_{jk}!}\right]\;\text{for}\;j,k=1,2,3=\frac{{\left(\gamma_{12}\left(t_{1}\right)\right)}^{n_{12}}\text{exp}\left(-\gamma_{12}\left(t_{1}\right)\right)}{n_{12}!}\times \frac{{\left(\gamma_{13}\left(t_{1}\right)\right)}^{n_{13}}\text{exp}\left(-\gamma_{13}\left(t_{1}\right)\right)}{n_{13}!}\times \frac{{\left(\gamma_{21}\left(t_{2}\right)\right)}^{n_{21}}\text{exp}\left(-\gamma_{21}\left(t_{2}\right)\right)}{n_{21}!}\times \frac{{\left(\gamma_{23}\left(t_{2}\right)\right)}^{n_{23}}\text{exp}\left(-\gamma_{23}\left(t_{2}\right)\right)}{n_{23}!}$$

Treating the factorial part in the equation as constant, taking logs both sides and re-arranging these terms yields

(3) $$\begin{array}{l} InL\left(\gamma_{jk}\left(t_{j}\right)\right)\propto n_{12}\ln\left(\gamma_{12}\left(t_{1}\right)\right)+n_{13}\ln\left(\gamma_{13}\left(t_{1}\right)\right)-\left[\gamma_{12}\left(t_{1}\right)+\gamma_{13}\left(t_{1}\right)\right]\\ \;+n_{21}\ln\left(\gamma_{21}\left(t_{2}\right)\right)+n_{23}\ln\left(\gamma_{23}\left(t_{2}\right)\right)-\left[\gamma_{21}\left(t_{2}\right)+\gamma_{23}\left(t_{2}\right)\right] \end{array}$$

The summation of transition rates from the same state is defined at the *flow rate, λ_j_*, which defines the probability of transition from state *j*. This means:

(4) $$\lambda_{j}=\sum_{j\neq k}\gamma_{jk}$$

Let *ρ_i,jk_* be the conditional probability that the next destination is state *k* given that the transition from state *j* to *k* occurs, the flow rate can be defined in terms of the conditional probability as:

(5) $$\gamma_{jk}=\{\begin{array}{l} \lambda_{j}\rho_{ik}\;\text{for}\;j\neq k\\ -\lambda_{j}\;\text{for}\;j=k\;\text{since}\;\rho_{jj}=\rho_{kk}=1 \end{array}$$

Substituting equation (4) expressions into equation (3), that is, *λ*_1_ (*t*_1_) = [*γ*_12_ (*t*_1_) + *γ*_13_ (*t*_1_)] and *λ*_2_ (*t*_2_) = [*γ*_21_ (*t*_2_) + *γ*_23_ (*t*_2_)] yields:

(6) $$InL\left(\gamma_{jk}\left(t_{j}\right)\right)=n_{12}\ln\left(\gamma_{12}\left(t_{1}\right)\right)+n_{13}\ln\left(\gamma_{13}\left(t_{1}\right)\right)-\lambda_{1}\left(t_{1}\right)+n_{21}\ln\left(\gamma_{21}\left(t_{2}\right)\right)+n_{23}\ln\left(\gamma_{23}\left(t_{2}\right)\right)-\lambda_{2}\left(t_{2}\right)$$

Differentiating equation (3) with respect to each transition rate and time (*γ_jk_* (*t_j_*)) in the equation, for instance, with respect to *γ*_12_ (*t*_1_) transition rate, yields:

(7) $$\frac{\partial InL\left(\gamma_{12}\left(t_{1}\right)\right)}{\partial \left(\gamma_{12}\left(t_{1}\right)\right)}=\frac{\partial }{\partial \left(\gamma_{12}\left(t_{1}\right)\right)}\left(n_{12}\ln\gamma_{12}\left(t_{1}\right)-\gamma_{12}\left(t_{1}\right)\right)=\frac{n_{12}}{\gamma_{12}\left(t_{1}\right)}-1$$

Equating the solution of equation (7) to zero and making the transition rate parameter the subject of formula yields a maximum likelihood estimate of the transition rate, that is,

(8) $${\hat{\gamma }}_{12}=\frac{n_{12}}{t_{1}}$$

Similarly, the other maximum likelihood estimates for the other transition rates would be:

(9) $${\hat{\gamma }}_{13}=\frac{n_{13}}{t_{1}};\;{\hat{\gamma }}_{21}=\frac{n_{21}}{t_{2}}\;\text{and}\;{\hat{\gamma }}_{23}=\frac{n_{23}}{t_{2}}$$

From this theoretical work, if one knows the number of transitions from state *j* to *k* which is *n_jk_* ; and the total exposure time in state *j* which is *t_j_*, then the transition rates can be estimated using equations (8) and (9). Similarly, if the waiting time in a particular state is unknown, that is, *t_j_*, the transition rate *γ_jk_* and the number of transitions *n_jk_* can give an estimate of the time is a specified state by re-arranging the same equations.

Due to the evolution of computational technology, multistate models from the MLE perspective are commonly implemented in R software(20) using the *msm* package(21). The *msm* package can fit continuous-time Markov models (homogeneous time or non-homogenous time using the piecewise constant models) and hidden Markov multistate models with misclassification error using individual-level data(3). Within the *msm* package, covariates can be included, survival graphs for each transition can be obtained, all data censoring types can be handled, the total length of stay in each state can be estimated, and model diagnostics can be done(3). In these models, the likelihood ratio test (LRT) is used to choose a better fit model between time-homogeneous and time non-homogeneous (piecewise constant) models(1). The goodness-of-fit (GOF) of the model is assessed by comparing the observed and the predicted number of individuals in each state at a specified time in a graph(1). The other test normally applied to all multistate Markov model processes, including those models with an absorbing state is the Pearson-type test which tests if the transition rates depend on several predictors(1). Additional information on other software packages which can handle multistate models is provided in Appendix 1.

### 2.2 The Bayesian Estimation (BE) Approach

The BE approach is a flexible method whose posterior transition estimates are based on the mapping of the prior distribution. The prior distribution constitutes the existing and new belief information for unknown parameters and the likelihood function of the observed data (22). This yields posterior estimates which are much more coherent compared to the MLE approach. Many different models can be fitted within the BE framework; however, the result validation is typically done through the prior sensitivity approach as many different priors can be tried (23). The nuisance priors within the BE modelling are always marginalised out of the joint posterior distributions, which is a straightforward way to deal with such parameters, and this is hardly done in the MLE approach (23).

In this section, we provide the theoretical aspects of BE regarding the Kolmogorov-Chapman forward equations. In general, the transition rates and transition probabilities are mapped using the Kolmogorov-Chapman forward equation, which has the following solution:

(10) $$P\left(t\right)=\exp\left(Q\left(t\right)\right)=\sum_{n=0}^{\infty }\frac{t^{n}}{n!}Q^{n}=\sum_{n=0}^{\infty }\frac{{\left(Q\left(t\right)\right)}^{n}}{n!}$$

where *P*(*t*) is the transition probability matrix, *Q*(*t*) is the transition rate matrix, 𝓃 defines the number of observed transitions and *t* defines the time. Let *γ_jk_* be the transition rate elements within the 3x3 matrix, let the *flow rate* from a defined state *j* be *λ_j_*, for instance, (*λ_1_* = *γ*_12_ + *γ*_13_) as previously described. Therefore, the transition rate matrix is defined as:

$$Q\left(t\right)=\left(\begin{array}{ccc} -\left(\gamma_{12}+\gamma_{13}\right) & \gamma_{12} & \gamma_{13}\\ \gamma_{21} & -\left(\gamma_{21}+\gamma_{23}\right) & \gamma_{23}\\ 0 & 0 & 0 \end{array}\right)=\left(\begin{array}{ccc} -\lambda_{1} & \gamma_{12} & \gamma_{13}\\ \gamma_{21} & -\lambda_{2} & \gamma_{23}\\ 0 & 0 & 0 \end{array}\right).$$

whose row totals sum to 0. Since state 3 is the absorbing state, the last row entries are equal to zero. With the *Q* (*t*) matrix, the Kolmogorov-Chapman forward equations solutions defined in equation (10) can be used to map the *P* (*t*) matrix which has the transition probability *π_jk_* (*t*) elements, that is,

$$P\left(t\right)=\left(\begin{array}{ccc} \pi_{11}\left(t\right) & \pi_{12}\left(t\right) & \pi_{13}\left(t\right)\\ \pi_{21}\left(t\right) & \pi_{22}\left(t\right) & \pi_{23}\left(t\right)\\ 0 & 0 & 1 \end{array}\right)$$

where the row totals add to 1. To fully define each transition probability *π_jk_* (*t*) component of the matrix, we modified slightly the solutions given elsewhere (24) to get comparable estimates to those obtained in R using *msm* library, assuming one has individually observed data. We considered this as a sensitivity approach to validate proper transcription of the code in WinBUGS. Let $h=\sqrt{{\left(\lambda_{1}-\lambda_{2}\right)}^{2}+4\gamma_{12}\gamma_{21}}$ for *λ*_1_ = *γ*_12_ + *γ*_13_ and *λ*_2_ = *γ*_21_ + *γ*_23_ and $e1=\text{exp}\left(-\frac{1}{2}\left(\lambda_{1}+\lambda_{2}-h\right)\times \left(2\times t\right)\right)\;\text{and}\;e2=\text{exp}\left(-\frac{1}{2}\left(\lambda_{1}+\lambda_{2}+h\right)\times \left(2\times t\right)\right).$ Then the Kolmogorov-Chapman solution for each transition probability *π_jk_* (*t*) element simplifies to:

(11) $$\begin{array}{l} \pi_{11}\left(t\right)=\frac{e1\times \left(-\lambda_{1}+\lambda_{2}+h\right)+e2\left(\lambda_{1}-\lambda_{2}+h\right)}{2h}\\ \pi_{12}\left(t\right)=\frac{\left(-\lambda_{1}+\lambda_{2}+h\right)\times \left(\lambda_{1}-\lambda_{2}+h\right)\times \left(e1-e2\right)}{4h\gamma_{21}}\\ \pi_{13}\left(t\right)=1-\pi_{11}\left(t\right)-\pi_{12}\left(t\right)\\ \pi_{21}\left(t\right)=\frac{\gamma_{21}\left(e1-e2\right)}{h}\\ \pi_{22}\left(t\right)=\frac{e1\times \left(\lambda_{1}-\lambda_{2}+h\right)+e2\times \left(-\lambda_{1}+\lambda_{2}+h\right)}{2h}\\ \pi_{23}\left(t\right)=1-\pi_{21}\left(t\right)-\pi_{22}\left(t\right) \end{array}$$

Let *n_jk_* be the number of transitions from state *j* to *k* for *j* ≠ *k*. If the data is partially observed over two points and intermediate transitions are unknown, then *n_jk_* is equal to the number of individuals since each individual contributes a single transition. In some instances, the data summaries or aggregated data may be available; and no individual information is available, then this modelling approach becomes relevant to use. The likelihood function of the number of transitions (*n_jk_*) can assume a multinomial distribution with probability parameters:

(12) $$\left(n_{j,1},n_{j,2},\ldots ,n_{j,m}\right)∼\text{Multinomial}\left(\pi_{j,1},\pi_{j,2},\ldots ,\pi_{j,m};n_{j}\right)$$

Since this is a full BE approach, the next step is to assign the prior information for the unknown transition rates parameters. These priors can either be informative or non-informative. Non-informative priors have little impact on the posterior distribution making the data (likelihood distribution) contribute more to the posterior estimates. Normally, non-informative priors are attained by using dispersed variance parameters or very small shape parameter values for the exponential rate parameter. Informative priors express exact and definite information about the unknown parameters being estimated. For such, prior information may be drawn from existing literature; however, the use of informative priors always ignites the prior subjectivity debate in BE. In this study, we assigned non-informative the priors for the unknown transition rate parameters (*γ*_12_, *γ*_13_, *γ*_21_ and *γ*_23_) to get comparable estimates to the MLE. Since the transition rates are from an exponential family distribution, we considered an exponential distribution with a small parameter value *β* = 0.001. The exponential probability density function is expressed as:

(13) $$P\left(\gamma_{jk}\right)\propto \beta \exp\left(-\beta \gamma_{jk}\right)$$

We further performed a sensitivity analysis of the exponential priors to assess if the choice of the prior distribution does not influence the transition rates estimated. A Weibull distribution prior with a scale of 0.001 and shape of 0.1; and a Gamma distribution prior with shape and scale of 0.1 were used to define the unknown transition rates priors. Since the shape and scale parameter values for Weibull and Gamma distributions were specified, we did not have any hyper-priors to specify further.

The Deviance Information Criterion (DIC) (25) was used to determine which model was a better fit after varying the prior information. To calculate the DIC value, the Markov chain Monte Carlo (MCMC) samples and the likelihood function of the observed data are required. The DIC is defined as:

(14) $$DIC=D\left(\bar{\theta }\right)+2p_{D}=\bar{D\theta }+p_{D}$$

where *θ* is the vector of all transition rate parameters, $D(\bar{\theta })$ is the deviance of the model evaluated at the posterior mean estimate $\bar{\theta }$ after averaging all MCMC samples of *θ*, $\bar{D\theta }$ is the average of Bayesian deviance or the posterior mean, *Dθ*, for all MCMC samples of *θ*. The unstandardized Bayesian deviance is defined by *Dθ* = −2log (*f* (*X* | *θ*)) where *f* (*x* | *θ*) is the likelihood function of the observed data *X* given the parameter *θ*. The quantity *p_D_* is the effective number of parameters, and is defined as $p_{D}=\bar{D\theta }-D(\bar{\theta }),$ where $\bar{\theta }$ is the average of MCMC samples of *θ*. The model with the least DIC value is the preferred model.

The posterior distribution fitted is given by equation (15) below, which reflects a joint distribution of the multinomial likelihood and the exponential prior distributions.

(15) $$P\left(\pi_{jk},\gamma_{jk}/n_{j}\right)\propto \left[\frac{\text{Γ}\left(\sum_{j}n_{j}+1\right)}{\prod_{j}\text{Γ}\left(n_{j}+1\right)}\prod_{j=1}^{m}\pi_{jk}n_{j}\right]\times \left[\beta \exp\left(-\beta \gamma_{jk}\right)\right]$$

Therefore, in this regard, the BE can be viewed as being a complete, unambiguous, prescriptive and coherent method of estimation as it combined both the likelihood information and the prior information to get the posterior estimates.

### 2.3 Multistate models assumptions

**Figure 3:**
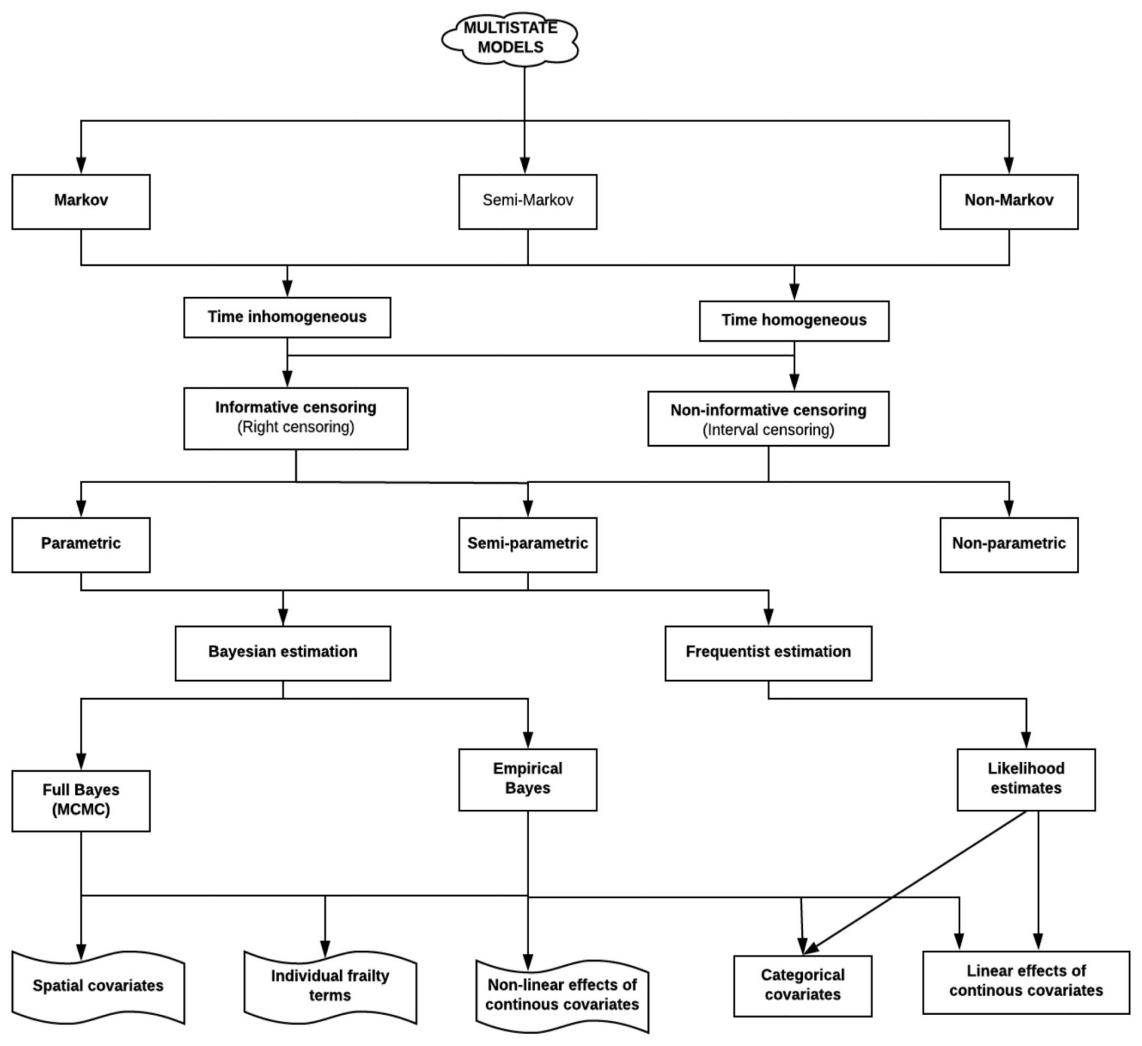
The multistate model assumptions, estimation types and possible covariates flow chart

The model assumptions behind these modelling approaches in these two packages overlap as both can assume the Markov process (assessed using the *markovchain* library in R), time homogenous transition rates (normally default set in most analysis packages) (26), and right censoring of observations. A flow chart for multistate model assumptions, methods of estimation, possible censoring patterns and covariates types is shown in Figure 3.

Multistate models can either be fitted assuming *discrete-time or continuous-time*. In discrete-time models, the movements between states occur at a fixed time, and the transition probabilities are usually reported. In contrast, in the stochastic continuous-time models, the transitions can occur at any time point, and these model inferences are usually based on the estimation of hazard rates or transition intensities (27). These transition rates describe the instantaneous rates at which the continuous-time multistate model transitions between states. In biomedical studies **of naturally occurring phenomena,** the continuous-time model reflects reality since the transitions occur at random.

Different assumptions can be made on multistate models about the dependency of the hazard rates (transition intensities) on time. The *Markov property* assumes that the transition to a future state is only dependent on the present state occupied not the ones before; hence, the model has a “memory loss”. This type of model is usually used because of its simplicity (28). Alternatively, multistate models can assume a semi-Markov process meaning that the next future transition depends on both the currently occupied state and also the time of entry into the current state. The *semi-Markov model* is considered flexible in most cases; however, there are some drawbacks in using this model. Firstly, the semi-Markov model contains many parameters which make the model much more problematic to fit and the distribution of the sojourn times in each state is a requirement which in most instances might be unavailable(4,15,29,30). Lastly, multistate models can assume a non-Markovian process. This model is dependent arbitrarily on the previously occupied states; hence, there is no “memory loss” in the model. The implementation of *non-Markovian models* has been challenging until the introduction of the “Markov-free” estimators for transition probabilities in the last decade(1).

Another assumption normally made in the multistate model is that of *time homogeneity.* In a time-homogeneous model, the transition intensities are assumed to be constant over time; that is, the rates are independent of time(1,29,31). In such models, the Kolmogorov differential equations can be solved explicitly using the decomposition of the transition matrix into both eigenvalues and eigenvectors(27). Models which assume time homogeneity are used more often possibly due to well-developed software at disposal and their less intimidating theoretical framework. However, if time homogeneity assumption is violated, an inhomogeneous time model is used which assume that the transition intensities change with time(31).

### 2.4 Multistate models data features and contrasts between BE and MLE

Multistate models can be characterised by the way the data have been captured in a research process. Censoring is a crucial feature in time-to-event data analysis(32).In observational studies, follow-up studies often end before the outcome occurs, leading to right-censoring of observation times(33). At the same time, left-censoring occurs when the study begins after the event has occurred, but the event times are unknown(32). Frequently, non-informative censoring occurs when participants are followed up intermittently such that the period between visits is missing. This means that the transition times are not precisely observed, and the states occupied between follow-up time points are unknown. These non-informative censoring observations are considered to be interval-censored(32). A special case of interval censoring is grouped survival data, in which the intervals for the subjects are completely identical or non-overlapping. Grouped survival data have been documented to be simpler to analyse (34). The mechanisms which give rise to censoring are essential in statistical inference within the multistate framework, and these data features need to be taken into account during analysis to avoid getting biased estimates since their likelihood functions will be different(35).

Moreover, intermittent follow-up of participants leads to incomplete spaced data points or missing information as a result of incomplete disease history of participants. In intermittent follow-ups, participants are observed for a short time, not to completion of their disease history as other visits are missed, and the specific time of occurrence of an event is usually unknown. The missing data in longitudinal studies and programmatic data frequently occur with different missing mechanisms to define the process. Missing data may be missing completely at random (MCAR) if the probability of missingness is not dependent on the unobserved or observed data. Suppose a study is comparing time to change of viral load level after ART initiation and the follow-up viral load values were not measured on some individuals because they transferred to another health facility. These missing viral load values may be considered to be MCAR if the decision by participants to move is not related to any variables considered in the running study. Missing at random (MAR) occurs if the probability that data are missing does not depend on unobserved data but may depend on observed data. Suppose that individuals who leave the study (become loss to follow-up (LTFU) and never return) are those with severe illness (high viral load), it is unlikely that the missing viral load measurements are MCAR, but the missing may be due to severely immune-deterioration of the patients; hence, the data is MAR. In contrary, if the patients who are severely immune deteriorating do not have viral load follow-up measurements because of their severity, the missing viral load values would not be MAR but missing not at random (MNAR). For such missing data mechanism, the reason for missingness has to be accounted for in the model to obtain precise results.

In practice, it is a challenge to distinguish the MAR mechanism from MNAR mechanism; therefore, a sensitivity analysis is encouraged to compare estimated of analysis under various missing data models to verify the stability of the inference. The multistate model framework allows one to use the likelihood-based method for MAR or MNAR covariates, assuming a continuous-time Markov multistate model(36). Work by Kalbfleisch and Lawless equips one to fit a time-homogeneous Markov model with arbitrary transitions structure for such incomplete history data(36). However, if any covariates are missing, convergence problems are more likely with this method; hence, most studies prefer to work with fully observed complete case data.

Multistate models can handle various types of covariates depending on the modelling approach used and the software the model is implemented in. Both methods of estimation can handle parametric effects of fixed covariates or categorical variables to assess their influence on the estimated transition processes(37). The MLE multistate models fitted within R *msm* library assume that the functional form of the effects of predictive factors is linear by default (or log-linear) which might not be the case always. This precludes the assessment of non-linear effects on the transitions rates using the MLE models. The violation of this assumption may lead to inaccurate statistical conclusions, the increased bias in estimates and decreases statistical power for statistical significance tests in MLE(3). In contrast, the BE multistate models within BayesX using the *bayesreg* object (38) can handle non-linear covariates effects of continuous covariates using penalised splines through their flexible predictor component(37). Besides, BE multistate models can handle time-varying effects and nonparametric baseline effects using penalised splines, which MLE multistate models cannot do.

Another strength of the BE multistate models over MLE models is their ability to account for frailty (random-effects) terms to explain unobserved heterogeneity in the collected information either at the individual level or spatial level which R *msm* library cannot do (37). For instance, in HIV disease progression model, it is vital to account for individual-level heterogeneity to estimate transition rates since patients respond differently to ART treatment, and these transitions may also vary by location. BE multistate models with spatial random effects were proposed by Kneib *et al.* (2008) with a demonstration on human sleep data(39). Their application to disease progression studies has been on the rise with recent work involving their application to HIV disease progression based on viral suppression and viral rebound model(40). This is one of the first papers to include the spatial effects within BE multistate models framework.

Moreover, in longitudinal studies with intermitted follow-up visits of participants, the number of visits may vary across participants. In such scenarios, the first visit (baseline) and the last observed visit assessment points (end of study) can be used. Such a data capturing process results in partially observed data which can be implemented within a multistate model framework. In such a scenario, only the initial state (baseline) and the last state (end of study) information in known but the intermediate experiences of an individual are unknown (or might not have been recorded). This happens typically in programme data where the data is usually reported and summarised in an aggregated format in national-level reports whereby detailed information of intermediate processes is usually missing or clinical trials whereby only the clinical endpoints are reported. Nonetheless, this data can be used within a multistate framework using the method proposed by Welton (2005) on handling partially observed aggregated data to estimate transition rates using Kolmogorov-Chapman forward equations(24). Such modelling approach of partially observed data provides a useful step in the research agenda as it forms the basis of future research formulation. This is one of the strengths of BE multistate models, which is not easily implemented in the MLE multistate models.

However, there is still under-utilised of BE approach in epidemiological studies though BE multistate models are much more flexible compared to MLE. BE multistate models can handle most of the data features like aggregated data which MLE multistate models cannot handle. The BE multistate models based on Kolmogorov-Chapman forward equations modelling approach using partially observed data forms the basis of this manuscript and the application details are outlined in the subsequent section.

## 3 Use of Kolmogorov-Chapman forward equations in Bayesian multistate models

In this application, we implemented the Kolmogorov-Chapman forward equations explained in section 2.2 on partially observed aggregated data to estimate transition rates using data from the Zimbabwe National ART programme (41). Viral load data were aggregated over a year from ART patients whose information was linked to the ePMS (42).

Following the WHO guidelines, individuals who are initiated on ART are monitored over time using CD4 cell counts and viral load to assess the regimen efficacy over time (43). In this example, the main focus was on using viral load measurements taken at baseline of ART initiation and repeatedly after 6 months. We extracted patient’s information spanning over a one year cycle of follow-up. We assumed that if individuals are initiated on potent and effective ART regimen and are adhering on treatment, after a year, a significant improvement in the immune response can be observed(44). It is based on this dataset that motivated our choice of model for illustration, which falls under disease progression models in Figure 1C.

In this study, we have data for individuals who had viral load measurements at baseline (at ART initiation) and one year after ART initiation. The in-between viral load clinical information was unknown since the data was partially observed with only two-time points. Since the data came from a routine ART programme setting, it is important to acknowledge that the period in which the data was captured was when differential monitoring was done(45,46); hence, few ART patients had their viral load measurement taken, and participant selection bias cannot be overlooked. Therefore, our final sample size had 5,596 participants with two observations taken at ART initiation (baseline) and after one year on ART.

For our illustration, a three-stage reversible multistate model, with states defined based on viral load measurements, was considered, and a schematic presentation of the diagram is shown in Figure 2. In this model, we assumed that individuals from state 1 (VL<50copies/uL) with an undetectable viral load may die (state 3) via state 2 (VL≥50copies/uL) which indicates a viral load rebound or detectable viral load, or directly from state 1 (VL<50copies/uL). Again individuals may move back to state 1(VL<50copies/uL) once they are in state 2 (VL≥50copies/uL) after ART which shows a reversible transition. We also assumed a time-homogeneous Markov model and the data were aggregated having been observed over two-time points in a single year follow-up. Since the data was partially observed, this means, the initial state at ART initiation and the final states after one year were known, but the route to the last observed state was unknown. In other terms, the intermediate transitions (viral load patterns) which occurred between the initial state and the last observed states are unknown. That is, individuals with undetectable viral load (state 1) at time 0 who later died after a year may have died directly from the state 1 (undetectable viral load) or may have died via state 2 (viral load rebound). The used partially observed aggregated vial load data is displayed in Table 1.

The total number of participants was 5,596 with 2,490 (44.5%) with an undetectable viral load (state 1) while 3,106 (55.5%) had a viral load rebound above 50copies/uL (state 2). At the end of one year, 165 (2.95%) participants had died of which 87 (53%) were initially in state 2 (viral load rebound) at baseline. Majority of the participants remained in their initial state; hence, assumed not to have made any transition out of their initially observed state.

Guided by the set of equations described in section 2.2, we implemented our model in WinBUGS software based on Gibbs sampling estimation. We estimated the transition rates using the partially observed aggregated data captured over a cycle of one year, as explained earlier. We set 15,000 MCMC simulations, a burn-in period of 1,000 simulations and thinning of 10. The code used is provided in Appendix 2, and this code may be viewed as a transcription of *P*(*t*) matrix each element fully defined (Equation 11), the likelihood function (equation 12) and different prior functions considered. A further sensitivity analysis to validate the transcription of the WinBUGS code was done using R *msm* package, and the used R code is provided in Appendix 2. The MLE and BE posterior estimates for transition rates are shown in Table 2.

Since the data were partially observed, there was uncertainty as to the exact route, an individual who reached state 3 (death) followed. However, there are four possibilities to describe this:

1. Participant arrived directly from state 1 having not visited state 2 during the follow-up period (state 1 to state 3).
2. Participant arrived directly from state 2 having not visited state 1 during the follow-up period (state 2 to state 3).
3. Participant arrived via state 2 from state 1 (state 1 to state 2 to state 3).
4. Participant arrived via state 1 from state 2 (state 2 to state 1 to state 3).

**Figure 4:**
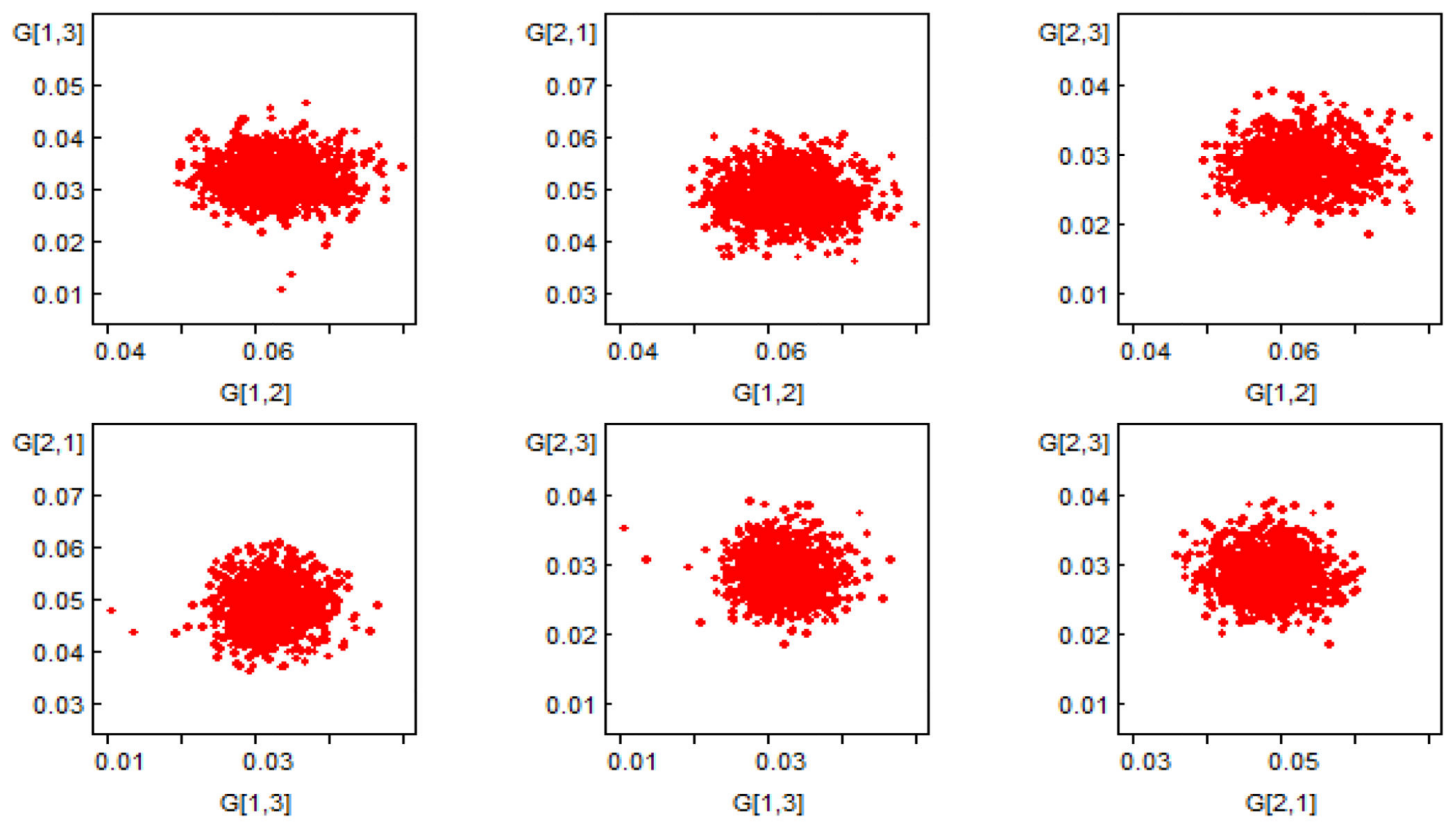
Bivariate scatter plots rates for the four transition rates parameters from the partially observed data

When we compared the model estimates from the MLE approach and the BE approach, both methods gave comparable transition estimates. However, the posterior transition from state 1 (VL<50copies/uL) to state 2 (VL≥50copies/uL) was 0.0633 (95% incredible interval (CI): 0.053-0.074). Movement from state 1 (VL<50copies/uL) to state 2 (VL≥50copies/uL) was 1.29 times (95%CI: 1.03-1.63) more likely than the transition from state 2 (VL≥50copies/uL) to state 1(VL<50copies/uL). Similarly, participants initially in state 1 (VL<50copies/uL) were 13% (1.1.31 95%CI: 0.81-1.59) more likely to die compared to those in state 2 (VL≥50copies/uL); however, this was not statistically significant. Those participants initially in state 1 (VL<50copies/uL) were 1.98 times (95%CI: 1.48-2.65) more likely to have a viral rebound compared to have died after 1 year while those participants initially in state 2 (VL≥50copies/uL) were 1.73 times (95%CI: 1.29-2.29) to have attained viral suppression to undetectable levels compared to have died. The correlations between the transition rates were positive, which means that the data on reaching state 3 (death) is compatible with an increase in each of the transition. This is also evident in the bivariate scatter plots for these four transitions in Figure 4.

The transition probabilities are shown in Table 3 for the three-cycle times, 3 months, 6 months and 1years cycles for both the BE approach and the MLE approach.

Similarly, the transition probabilities were similar for both the MLE and the BE approaches. From these estimates, the probability of leaving the initial state increases as the cycle time increases, that is, for a transition from state 1 (VL<50copies/uL) to state 2 (VL≥50copies/uL), the probability at 3 months cycle was smaller than the probability at 6 months cycles, and both were smaller than the probability at 1-year cycle: (*π*_12_(*t* = *3months*) =0.0155<*π*_12_ (*t* = 6*months*) =0.0302<*π*_12_ (*t* = 1*year*) =0.058). This pattern was similar across other transitions and was as anticipated for such types of models.

## 4 Discussion

In this manuscript, we have highlighted the usefulness of multistate models as they account for the time change, and individuals multiple outcomes can be estimated simultaneously in epidemiological studies compared to the traditional survival models. We have also discussed two methods of statistical inference with emphasis on the BE approach, which has not been fully utilised and reviewed in the literature. Various types of multistate models disposable to use in different science fields to answer different kinds of research questions from longitudinal time to event data have been outlined. Multistate model assumptions and data featured have also been highlighted. Finally, an application of the Kolmogorov-Chapman forward equation in estimating transition rates has been demonstrated using partially observed aggregated viral load data for the first time in HIV disease progression modelling. The application model estimates were comparable to existing epidemiological work on viral transitions patterns(40,44).

This manuscript put forward the advantage of using multistate modelling as this approach may bring out new and important biological insights in understanding the intermediate processed of a disease which ordinary regression models like Cox proportional hazard model may be ignoring. Multistate models’ transition processes are easy to understand as they can be drawn into schematic diagrams of mutually exclusive states which help to understand the model better. Prediction of an individual’s disease progression trajectories through time is another advantage of multistate models, and this is helpful for programme managers to make informed decisions which traditional survival models cannot do.

This manuscript further put forward the strengths of BE multistate models over MLE multistate models. It is undebatable that individual-level data is much for informative and provides more extensive flexibility in modelling. However, aggregated data usually is accessible on many public platforms and intermediate observation times during follow-up studies may not always be supported in most programs due to financial constraints. In such instances, partially observed aggregated data becomes readily available and disposable to everyone without requiring much stringent permission to use it. Moreover, clinical trials studies may also report partially observed time to clinical outcomes (endpoints) data without reporting any intermediate states (47). This motivated the example application provided in this manuscript, as it emphasised the use of BE multistate model in handling partially observed aggregated data using the Kolmogorov-Chapman forward equations on a new dataset on viral load measurement in HIV studies. Our application also put forward how partially observed data in principle can be used in an epidemiologically realist model(47). Handling of such aggregated data is precluded within MLE multistate models implemented in R using *msm* package which only favours individual-level data. The ability of BE multistate models to handle aggregated data affirms how important aggregated data could be in instances where there is no individual-level data, and quick baseline information is required for a more informative hypothetical formulation for future researches.

The MLE approach generally relies on the available data defined through the maximum likelihood function; hence, it is generally affected by the small sample sizes of data(23). The small sample sizes may result in the inferential process to be internally logically inconsistent. In contrast, the BE approach integrates the likelihood function and the prior function, which contains existing information to give comprehensive posterior estimates of the transition rates, and this is an advantage of the BE approach. The addition of prior information in the BE approach allows the operating characteristics of the Bayesian methods in small sample sizes and overall, to be dependable. Bayesian methods tend to perform well with limited data, while frequentist MLE inference appeals to large sample theory and thus may break down with small sample sizes. This advantage is more evident if informative priors are used which have a strong impact on the posterior estimates compared to when non-informative priors are used since using non-informative priors mimic the MLE results.

Results interpretations of the MLE approach are customarily based on the repeated sampling property, which may be ambiguous; the probability of the hypothesis is non-applicable, unlike the BE approach which makes use of the probabilities for both the hypothesis and data, which is what is required precisely to make decisions (48). The direct probability statements in BE inference about the parameters is much more useful compared to the frequentist statistics. In other terms, BE inference provides fixed probability intervals for unknown quantities compared to the random intervals for fixed quantiles from the MLE inference. This makes the BE approach much more compelling than the MLE approach as it provides the kind of answers most researchers require. In most studies, the BE approach has proven to outperform the MLE approach (23).

As much as BE approach is theoretically simpler, more robust, more flexible and easier to implement with minimum support as compared to the MLE approach (47); the adoption and utilisation of the BE approach have been hindered by the difficulties faced by researchers in implementing the Bayes models. The main hindrance of Bayesian models is the prior specification choices, which is normally subjective. This is quite a debatable issue in Bayesian modelling since the observed posterior estimates are heavily dependent on the prior specification, especially for informative prior choices. However, to override this argument, assigning diffuse (vague/non-informative) priors is encouraged, and results are comparable with the frequentist estimates(24,39). In other words, the BE models can be run with prior references which can mimic the MLE (non-informative priors) as compared to the use of informative priors (23). This point can be validated by our posterior transition rates estimates which were similar to those estimated through the MLE using R *msm* package since we used purely non-informative priors in our modelling approach. This similarity puts forwards that even if aggregated data is used, the findings are comparable to the MLE and decisions can be informed accordingly. Also, prior sensitivity analysis is encouraged as an excellent modelling courtesy to validate that the choice of the prior used did not influence the results obtained.

Although, BE models are computationally intensive requiring much longer running time of more hours or days to converge compared to the MLE multistate model(40). Still, MLE models may fail to converge if the models are complex of which model convergence can be only achieved through the BE route. This leaves the issue of longer running times invalid since the BE approach will be the only route to consider in such instances. Moreover, multistate models, particularly those fitted in BayesX using the *bayesreg* object have a limitation of model comparison techniques since the DIC cannot be estimated in such models(48). This favours the MLE, which uses Akaike’s Information Criterion (AIC) and LRT for model comparison to validate the obtained estimates.

Moreover, as much as the BE multistate model on aggregated data using Kolmogorov-Chapman differential equations in WinBUGS is useful in situations where individual-level data is not available, proper implementation of the model might be a constraint to other researchers as the modelling approach require technical expertise to achieve convergence in terms of correct model specification (transcription of the Kolmogorov-Chapman forward equations into a code). However, this hindrance can be solved by strengthening resource sharing between researchers through including codes in publications and other platforms. Another limitation of the BE multistate model in WinBUGS could be explicitly solving for the Kolmogorov’s forward equations solution to be able to transcribe the solutions into the model code. This exercise becomes hectic and tedious if the model has many model states, reversible transitions; hence, the model becomes complex. However, the WinBUGS Differential Interface (WinDiff) software can be used instead to mitigate such challenges(24).

As much as the use of Kolmogorov-Chapman forward equations on partially observed aggregated data could be helpful in epidemiological researches, it is important to note that this modelling approach has some limitations too. There are conditions under which an analysis of aggregated data (i.e., summary data over individuals) would be biased. Firstly, the method restricts attention to a situation whereby only two observations are made on an individual (at baseline and the end of study). One may think this approach violated the natural process of events in medical research as participants go through various intermediate processes between the stipulated times (one year cycle in this case) which are not incorporated in the model leading to biased estimates. However, by assuming that the observed states follow a multinomial distribution with respect to the initial state and the transition probability are depend on the total observation time lined through the Kolmogorov-Chapman forward equations leverages this problem. Also, with only a few observations, BE shows to be more sensitive to the prior distribution for parameters, and a lot of pressure for the posterior estimates is based on the correct prior choices.

Moreover, the other condition under which an analysis of aggregated data would be biased is that there is uncertainty as to the exact route, an individual who reached state 3 (death) followed. This uncertainty compromises the validity of the reported posterior estimates, which is what researchers in epidemiological studies are interested in. In our application, the fitted model assumed constant transition rates over time (time-homogeneous), an assumption well known to be unrealistic in many kinds of epidemiological researches. This assumption may result in biased estimates; hence, time-inhomogeneous transition rates should be considered for unbiased estimates. Lastly, this method needs to be improved so that its covariates effects on the aggregated transition rates can be accounted for.

In conclusion, multistate modelling offers a flexible tool in studying disease progression and estimating transition rates using various forms of assumptions, estimation methods and data features. Multistate models bring out significant disease progression understandings which the traditional naïve regression models may ignore. Hence, multistate models should be used as a supplement to the traditional naïve regression models to gain additional information. As much as the frequentist (MLE) approach continues to dominate research, particularly in life sciences, it is restrictive in providing comprehensive estimates based on assumptions which reflect reality in disease progression models. Therefore, this research study recommends the use of BE models which can implement assumptions reflect natural processes of events and use the probability of the hypothesis and data which most epidemiologists prefer. The use of the Kolmogorov-Chapman forward equations on aggregated data also helps to open the way to a more formal and systematic use of available literature like national reports or clinical trials to inform Markov models in decision making. However, it is crucial to understand the model development process which might be challenging and make use of the sensitivity analysis approach as there could be a problem of the surfeit of posterior distributions due to prior variations to validate the results in BE approach.

## Supplementary Material

Appendix

## Figures and Tables

**Table 1:** Partially observed data for viral load suppression among adult ART patients from the Zimbabwe national ART program after a year time cycle for a three-state model

			Final state after a year of follow-up, *k*		
	Number of participants at baseline	Initial state, *j*	State 1	State 2	State 3
	2490	State 1	2269	143	78
	3106	State 2	137	2882	87
	0	State 3	0	0	0
**Totals**	5596		2406	3025	165

**Table 2:** Posterior estimates and correlations for the transition rates for the three-state model using partially observed data from the adult ART patients in the Zimbabwe national ART data after a year of follow-up.

	Transition rate parameter estimates							
	MLE		BE					
Parameter	No prior information (AIC=3700.88)	Weibull priors (DIC=33.859)	Gamma priors (DIC=33.724)	Exponential priors (DIC=33.325)	BE Correlations			
	TR coefficient (95% CI)*	TR coefficient (95% CI)	TR coefficient (95% CI)	TR coefficient (95% CI)	*γ*_12_	*γ*_13_	*γ*_21_	*γ*_23_
*γ*_12_	0.06242 (0.053-0.074)	0.0625 (0.053-0.073)	0.0627 (0.053-0.075)	0.0633 (0.053 to 0.074)	1.0	0.068	0.124	0.0985
*γ*_13_	0.0315 (0.025-0.039)	0.0317 (0.024-0.041))	0.0319 (0.026-0.039	0.0323 (0.026 to 0.040)		1.0	0.121	0.046
*γ*_21_	0.0481 (0.041-0.051)	0.0478 (0.040-0.056)	0.048 (0.040-0.057)	0.0485 (0.041 to 0.056)			1.0	0.015
*γ*_23_	0.0279 (0.022-0.035)	0.0284 (0.0232-0.028)	0.028 (0.023-0.035)	0.0285 (0.023 to 0.035)				1.0

MLE=maximum likelihood estimation, BE=Bayesian estimation, TR=transition rate, CI=credible interval, CI*=confidence interval, AIC=Akaike’s Information criterion, DIC=Deviance Information Criterion

**Table 3:** Posterior estimates for the transition probabilities during three months, 6months and a one year cycle for the three-state model using partially observed data from the adult ART patients in the Zimbabwe national ART data after a year of follow-up.

	3 months		6 months		1 year	
	BE	MLE	BE	MLE	BE	MLE
**Parameter**	Transition rate estimate (95% CI)	Transition rate estimate (95% CI)	Transition rate estimate (95% CI)	Transition rate estimate (95% CI)	Transition rate estimate (95% CI)	Transition rate estimate (95% CI)
*π*_11_ (*t*)	0.9765 (0.973 to 0.980)	0.976877 (0.974 to 0.980)	0.9530 (0.945 to 0.960)	0.95447 (0.948 to 0.960)	0.9103 (0.898 to 0.922)	0.91170 (0.899 to 0.922)
*π*_12_ (*t*)	0.0155 (0.013 to 0.018)	0.015278 (0.013 to 0.018)	0.0302 (0.026 to 0.036)	0.02992 (0.025 to 0.035)	0.0580 (0.048 to 0.068)	0.05737 (0.049 to 0.067)
*π*_13_ (*t*)	0.0081 (0.006 to 0.010)	0.007845 (0.006 to 0.010)	0.0160 (0.012 to 0.020)	0.01562 (0.012 to 0.020)	0.0318 (0.026 to 0.039)	0.03093 (0.025 to 0.039)
*π*_21_ (*t*)	0.0119 (0.010 to 0.014)	0.011770 (0.010 to 0.014)	0.0231 (0.019 to 0.027)	0.02305 (0.019 to 0.027)	0.0442 (0.037 to 0.052)	0.04419 (0.037 to 0.052)
*π*_22_ (*t*)	0.9810 (0.979 to 0.983)	0.981280 (0.979 to 0.984)	0.9626 (0.958 to 0.967)	0.96309 (0.958 to 0.967)	0.9273 (0.918 to 0.936)	0.92823 (0.918 to 0.936)
*π*_23_ (*t*)	0.0071 (0.006 to 0.009)	0.006951 (0.006 to 0.009)	0.0142 (0.011 to 0.017)	0.01386 (0.011 to 0.017)	0.0285 (0.023 to 0.035)	0.02758 (0.023 to 0.034)

BE-Bayesian estimation; MLE-maximum likelihood estimation, CI-credible/confidence interv
