# Supplementary material for: A review of multistate modelling approaches in monitoring disease progression: Bayesian estimation using the Kolmogorov-Chapman forward equations
Source: Stat Methods Med Res. Author manuscript; Available in PMC 2022 Apr 15. (PMC7612622; doi:10.1177/0962280221997507)
Supplement: Appendix [file EMS144247-supplement-Appendix.pdf]

ZMZ conceptualised the paper, cleaned and analysed the data and drafted the manuscript**, JT** and **TC** reviewed the manuscript for intellectual content, **EM** guided the whole paper structure development. All authors reviewed the final manuscript for submission**.**

Conflict of Interest

The authors declare no competing interests for this manuscript.

Appendix 1: Statistical software and challenges

In addition to the use of *msm* package in R which can handle maximum likelihood estimation (MLE) multistate models, the other library within R software is the *tdc.msm* library developed by Meira-Machado *et al.* (2009) which can fit five different multistate models including time-homogeneous and non-homogeneous Markov multistate models; and Cox Markov and Cox semi-Markov multistate models (1). The *tdc.msm* library is a comprehensive package for modelling multistate longitudinal data since different models can be fitted within one library, and model comparison can be done easily (1). For nonparametric estimation, the *msSurv* library within R can be used to estimate state occupation probabilities, initial and exiting time in a state, and the marginal integrated transition rate for the non-Markov multistate process (49). The other R package is the *mstate* developed by Wreede *et al*. (2011) for both the competing risk models and multistate models (50). Additional R libraries are the *etm* library Allignol *et al*. (2011) for empirical transition probabilities and the *changeLOS* (change length of hospital stay) library introduced by Wangler *et al*. 2006) for the Aalen-Johansen Estimator is implemented within R software. The limitation of the *changeLOS* library is that it does not support the inclusion of covariates in the multistate model and left truncated data (51). However, the *mvna* library can handle both left truncated and right censored multistate data (51).

The STATA software (licensed for use) (52) can fit the MLE multistate models using the *multistate model ado files* developed by Cowther and Lambert (2016) which restructures and declares the multistate data as survival and any survival model within STATA can be used (53). This package can estimate each transition rate by its unique model structure, assuming either a Markov or semi-Markov process (53). Uniquely to the STATA models is the ability to estimate each transition rate assuming different hazard functions which best fit the transition as compared to the R *msm* models which assumes the same hazard function on all the model transition processes. Another option in STATA is using *illdprep* and *stpm2illd* commands which can perform a similar analysis as described elsewhere (54).

The Bayesian estimation (BE) multistate models can be implemented in BayesX software using the *bayesreg* object. This package can handle several data features which the MLE cannot handle like non-linear effects of continuous covariates, time-varying effects, adjust for individual and spatial random effects accounting for unobserved heterogeneity. The WINBUGS software which estimates using Gibbs Sampling also can handle BE multistate model through the use of Kolmogorov-Chapman forward equations. This modelling approach promotes the use of partially observed aggregated data which the MLE approach cannot do as illustrated in the main text.

Most of these statistical packages which can handle multistate models, both from the MLE and BE are free while few may require licensing. Here we highlight a couple of strengths and limitations associated with each software type which handles multistate models, some of which might not have been discussed in detail in this manuscript. Firstly, most of the existing software assumes the Markov property and time-homogeneous by default which makes it difficult if these assumptions are violated. Secondly, the Markov assumption can be difficult to test, and in most cases, studies are silent on pre-model assumption testing of the Markov property; however, *markovchain* library implemented in R is one of the packages which can test for Markov property (26). Thirdly, not all software types are freely available for use like BayesX,

WinBUGS and R, some of the software types require a license to be granted access, for instance, STATA and SAS. Fourthly, data argumentation (structure) is different in each of the software, which may be a major drawback if one wishes to make a comparison across the software. In addition to this, at times convergence is an issue in multistate models depending on the size of the data, the model complexity of the proposed model (number of states, reversible transition and number of covariates included) and the preferred method of estimation (BE or MLE) as the models may take much longer (hours or days) to converge or may fail to converge at all. Lastly and most importantly, the result outputs vary moving from one software package to another; hence, methodological background theory for each package is crucial to enable results comparison. For instance, in the R *msm* library, one gets the exact transition rate values which can be interpreted directly while in BayesX outputs, the estimates are based on the flexible predictor on a log scale (39).

Appendix 2: WinBUGs three state reversible multistate model code

###MODEL

```
model {
#####Multinomial likelihood for observed data
        for (i in 1:2)
            {
            r[i,1:3] ~ dmulti(P[i,1:3],n[i])
            }
#####Find transition probabilities (for given time) in terms of rates

h<- sqrt(pow(lambda[1]-lambda[2], 2) + 4*G[1,2]*G[2,1])
e1<-exp(-.5*(lambda[1] + lambda[2] - h)*(2*t.obs))
e2<-exp(-.5*(lambda[1] + lambda[2] + h)*(2*t.obs))

P[1,1]<-((-lambda[1]+lambda[2]+h)*e1+ (lambda[1]-lambda[2]+h)*e2)/(2*h)
P[1,2]<-((-lambda[1]+lambda[2]+h)*(lambda[1]-lambda[2]+h)*(e1-e2))/(4*h*G[2,1]) P[1,3]<- 1- P[1,1] - P[1,2]
P[2,1]<- G[2,1]*(e1-e2)/h
P[2,2]<- ((lambda[1]-lambda[2]+h)*e1+ (-lambda[1] + lambda[2] + h)*e2)/(2*h)
P[2,3]<-1 - P[2,1] - P[2,2]

#Give exponential priors for unknown transition rate parameters
        for (i in 1:2)
           {
        for (j in (i+1):3)
                    {
                    G[i,j] ~ dexp(.001)
                    }
           }
       for (i in 2:2)
          {
       for (j in 1:(i-1))
                   {
                  G[i,j] ~ dexp(.001)
                   }
           }
lambda[1]<- G[1,2] + G[1,3]
lambda[2]<- G[2,1] + G[2,3]

######Find P(t.new) for given new time of interest=1 years
e1.new<-exp(-.5*(lambda[1] + lambda[2] - h)*(2*t.new))
```

```
e2.new<-exp(-.5*(lambda[1] + lambda[2] + h)*(2*t.new))
Pt[1,1]<-((-lambda[1]+lambda[2]+h)*e1.new+ (lambda[1] -lambda[2]+h)*e2.new)/(2*h)
Pt[1,2]<-((-lambda[1]+lambda[2]+h)*(lambda[1]-lambda[2]+h)*(e1.new-e2.new))/(4*h*G[2,1])
Pt[1,3]<-  1-Pt[1,1] - Pt[1,2]
Pt[2,1]<- G[2,1]*(e1.new-e2.new)/h
Pt[2,2]<- ((lambda[1]-lambda[2]+h)*e1.new+ (-lambda[1] + lambda[2] + h)*e2.new)/(2*h)
Pt[2,3]<-  1-Pt[2,1] - Pt[2,2]
##### P(t.final)=half a year (put 0.25 in the data)
e1.final<-exp(-.5*(lambda[1] + lambda[2] - h)*(2*t.final))
e2.final<-exp(-.5*(lambda[1] + lambda[2] + h)*(2*t.final))
Ptn[1,1]<-((-lambda[1]+lambda[2]+h)*e1.final+ (lambda[1] -lambda[2]+h)*e2.final)/(2*h)
Ptn[1,2]<-((-lambda[1]+lambda[2]+h)*(lambda[1]-lambda[2]+h)*(e1.final-e2.final))/(4*h*G[2,1])
Ptn[1,3]<-1 - Ptn[1,1] - Ptn[1,2]
Ptn[2,1]<- G[2,1]*(e1.final-e2.final)/h
Ptn[2,2]<- ((lambda[1]-lambda[2]+h)*e1.final+ (-lambda[1] + lambda[2] + h)*e2.final)/(2*h)
Ptn[2,3]<-1 - Ptn[2,1] - Ptn[2,2]
}
```

###DATA

```
iist(r=structure(.Data=c(2269,143,78,137,2882,87),.Dim=c(2,3)),n=c(2490,3106),t.obs=0.5,                    t.new=0.25,
t.final=0.125)
```

###INITIAL VALUES

```
list(G=structure(.Data=c(NA,.1,.1,.1,NA,.1),.Dim=c(2,3)))
```

Here is an R code for comparable results if one has individual-level data

```
#StepVL1-is the name of the dataset as saving name
#ID-is the unique identifier
#States-is the variable which defines individual states
#Time1-if the cumulative time
library(haven)
StepVL1 <- read_stata("C:/Users/ WinBUGS/VL_KOL_1.dta")
statetable.msm(States, ID, data=StepVL1)
twoway3.q <- rbind(c(0.25, 0.5, 0.25), c(0.25, 0.5, 0.25), c(0, 0, 0))
crudeinits.msm(States ~ Time1, ID, data=StepVL1, qmatrix=twoway3.q)
StepVL1.msm <- msm( States ~ Time1, subject=ID, data = StepVL1,
                qmatrix = twoway3.q, deathexact = 3,
               control = list (fnscale = 5000, maxit= 500 ))
###To get the transition rates
StepVL1.msm
###To get the transition probabilities
P_1year<-pmatrix.msm(StepVL1.msm,t=1)
P_6months<-pmatrix.msm(StepVL1.msm,t=0.5)
P_3months<-pmatrix.msm(StepVL1.msm,t=0.25)
```

REFERENCES

1. Meira-Machado L, Uña-álvarez J De, Cadarso-suárez C, Andersen PK. Multi-state models for the analysis of time-to-event data. Statistical methods in Medical Research. 2009;18(2):195–222.

2. Hougaard P. Multi-state Models : A Review. Lifetime Data Analysis. 1999;264:239–64.
